# Multiple Metastases to the Lungs 14 Years After Surgery for Adult‐Type Granular Cell Tumor of the Ovary

**DOI:** 10.1002/kjm2.70032

**Published:** 2025-04-28

**Authors:** Di‐Ping Yu, Liu‐Qing Yang

**Affiliations:** ^1^ Department of Pathology The Puer People's Hospital Puer Yunnan Province China; ^2^ Department of Nephrology The Puer Hospital of Traditional Chinese Medicine Puer Yunnan Province China

Adult‐type granulosa cell tumor (AGCT) is a rare subtype of ovarian tumor originating from the mesenchymal component of the ovarian sex cord. The natural history of this disease is characterized by slow growth, local spread, and late recurrence [1]. Recurrence with pulmonary metastasis is rare. This article reports a case of a woman with FIGO IA stage ovarian AGCT with multifocal pulmonary metastases detected 14 years after initial diagnosis.

A 54‐year‐old female patient underwent total abdominal hysterectomy and bilateral salpingo‐oophorectomy for a FIGO IA stage ovarian AGCT in February 2007 and received no adjuvant chemotherapy. She did well until May 2021, when routine chest computed tomography (CT) revealed multiple bilateral pulmonary nodules with a maximum diameter of 1.4 cm (Figure 1A), accompanied by mildly elevated serum CA‐125 level of 47.7 U/mL (normal range: 0–35 U/mL). The patient underwent video‐assisted thoracoscopic surgery (VATS) to remove a lesion in the right lung. Histopathologic examination revealed a metastatic tumor with a clear boundary and a typical Call‐Exner body (Figure 1D,E), and immunohistochemical staining demonstrated a positive result for inhibin‐α, SF‐1, and FOXL2 (Figure 1F). Consequently, the pathological diagnosis was pulmonary metastatic ovarian AGCT. The patient started adjuvant chemotherapy with six cycles of paclitaxel and carboplatin in July 2021. The follow‐up was discontinued for 1 year, and the patient was not examined again until January 2023. The chest CT scan showed the tumors had substantially increased in size and number (Figure 1B). She continued to be treated for six cycles of paclitaxel and carboplatin. After completing the second cycle of chemotherapy, the follow‐up CT scan showed no significant changes in the tumor (Figure 1C). Unfortunately, serum anti‐Müllerian hormone (AMH) and inhibin B levels were not assessed, and the patient remained asymptomatic throughout. In the subsequent telephone follow‐up, the patient told us that she voluntarily chose to stop further chemotherapy due to financial difficulties. The patient reported no other physical discomforts and currently remains alive. We deeply regret the patient's decision.

**FIGURE 1:**
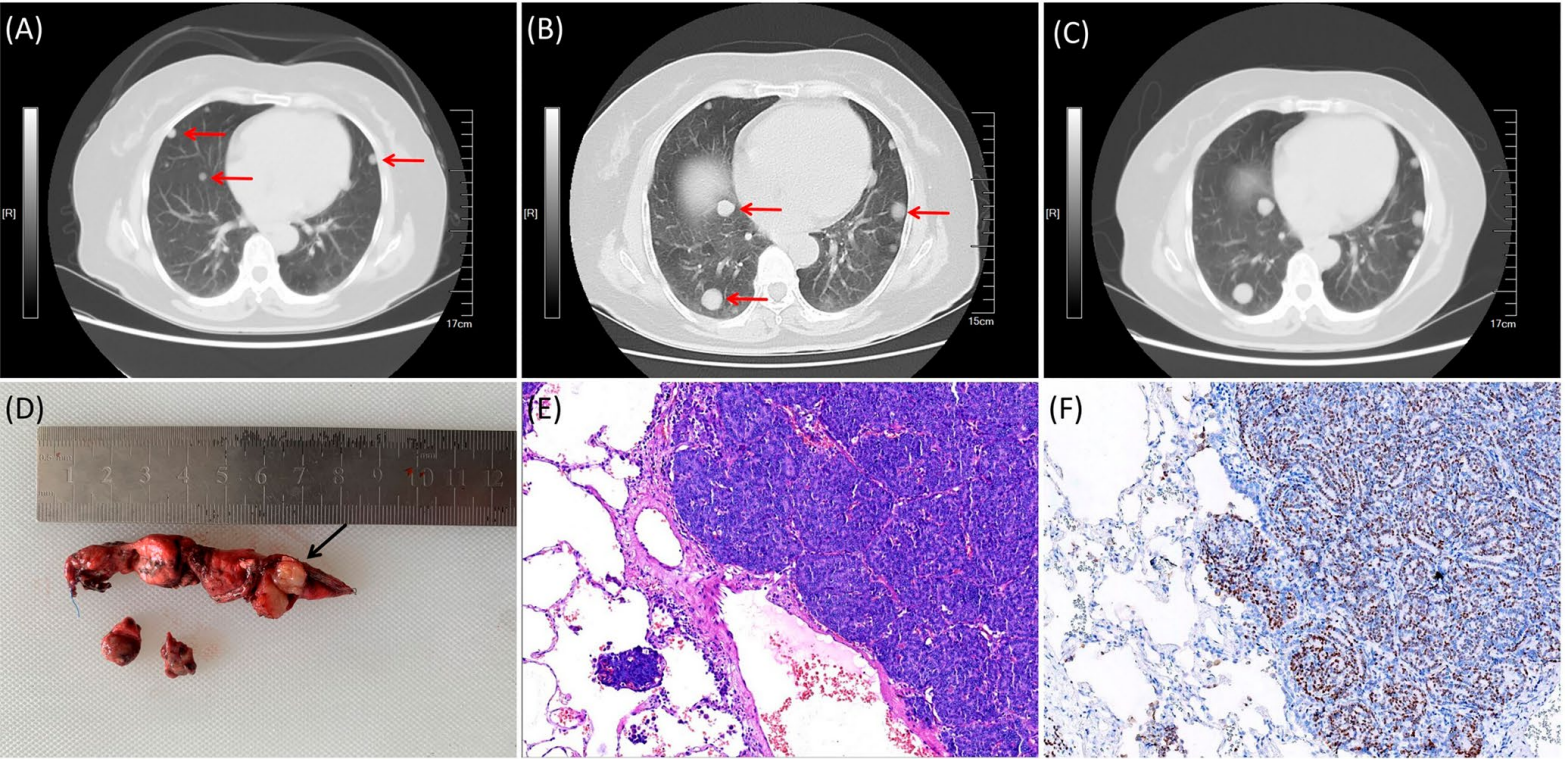
(A) Chest CT lung window revealed well‐defined multiple nodules (red arrow) in both lungs (May 2021). (B) Chest CT lung window that the size and number of tumors had greatly increased (red arrow) (January 2023). (C) The chest CT scan showed no significant changes in the tumor after the completion of second chemotherapy (July 2023). (D) Gross mrophological appearance of the tumor (black arrow). (E) Microscopic findings showing the structure of Call‐Exner bodies in the tumor tissue of the lung (H&E, original magnification × 40). (F) Immunohistochemical staining positive for FOXL2 (original magnification × 100).

Most patients with AGCT generally present with stage I disease (78%–91%), with the lesion being confined to the ovary. Only a few cases of pulmonary metastasis in stage I AGCT have been reported in the literature [1, 2, 3, 4], with one case having metastasis 36 years after treatment of the initial ovarian neoplasm [4]. All patients are asymptomatic at presentation, and metastasis is typically detected incidentally during routine follow‐up examinations. The most commonly utilized tumor markers for AGCT detection and recurrence are AMH and inhibin B. AMH is a specific circulating marker for AGCT. Its diagnostic performance appears to be effective for primary and recurrent AGCT, with 92% sensitivity and 81% specificity [5]. Serum CA‐125 concentration has been used to detect primary ovarian tumors and recurrences; however, a consistent correlation could not be confirmed between its levels and tumor activity [1]. A definitive diagnosis relies on pathological examination. The nuclear groove and Call‐Exner body are typical pathological features of AGCT, and immunohistochemical staining showed the expression of FOXL2, inhibin‐α, SF‐1, and calretinin.

There are no established clinical practice guidelines for the management of recurrent AGCT. However, treatment strategies such as surgical resection, chemotherapy, and radiation therapy are utilized clinically to manage recurrent AGCT. Moreover, surgical resection to eradicate the residual disease can improve a patient's postoperative quality of life and recurrence‐free survival.

In conclusion, AGCT is an ovarian tumor with a low malignant potential that is prone to late recurrence and multiple recurrences. This case illustrates the importance of long‐term follow‐up for early stage AGCT. AMH and inhibin B are important tumor markers for the detection of recurrence.

## Conflicts of Interest

The authors declare no conflicts of interest.

## Data Availability

Data sharing is not applicable to this article as no new data were created or analyzed in this study.
